# Single-Cell Chemical Lysis on Microfluidic Chips with Arrays of Microwells

**DOI:** 10.3390/s120100347

**Published:** 2011-12-30

**Authors:** Chun-Ping Jen, Ju-Hsiu Hsiao, Nikolay A. Maslov

**Affiliations:** 1 Department of Mechanical Engineering and Advanced Institute of Manufacturing with High-Tech Innovation, National Chung Cheng University, Chia Yi 62102, Taiwan; E-Mail: ggina03@yahoo.com.tw; 2 Khristianovich Institute of Theoretical and Applied Mechanics, Siberian Division, Russian Academy of Science, Novosibirsk 630090, Russia; E-Mail: nmaslov@itam.nsc.ru

**Keywords:** microwell, single-cell, cell lysis, microfluidics

## Abstract

Many conventional biochemical assays are performed using populations of cells to determine their quantitative biomolecular profiles. However, population averages do not reflect actual physiological processes in individual cells, which occur either on short time scales or nonsynchronously. Therefore, accurate analysis at the single-cell level has become a highly attractive tool for investigating cellular content. Microfluidic chips with arrays of microwells were developed for single-cell chemical lysis in the present study. The cellular occupancy in 30-μm-diameter microwells (91.45%) was higher than that in 20-μm-diameter microwells (83.19%) at an injection flow rate of 2.8 μL/min. However, most of the occupied 20-μm-diameter microwells contained individual cells. The results of chemical lysis experiments at the single-cell level indicate that cell membranes were gradually lysed as the lysis buffer was injected; they were fully lysed after 12 s. Single-cell chemical lysis was demonstrated in the proposed microfluidic chip, which is suitable for high-throughput cell lysis.

## 1. Introduction

Cell lysis is crucial in the analysis of intracellular components containing information about genetic or disease characteristics in genomics, proteomics, and metabolomics [1]. Many conventional biochemical assays are performed using populations of cells to determine their quantitative biomolecular profiles. However, population averages do not reflect actual physiological processes in individual cells, which occur either on short time scales (e.g., kinase signaling cascades) or nonsynchronously (e.g., response to an external chemical gradient) [2]. As a result, accurate analysis at the single-cell level has become a highly attractive tool for investigating cellular content.

Devices created using microfabrication technologies allow the precise manipulation of biological cells, and thus have the potential to provide individual characterization, lysis, detection, and assay of cells at the single-cell level. Microfabrication technologies combined with surface chemistry have stimulated research to understand the fundamental cell biology and pharmaceutical analysis by exposure of cells to drugs and environmental perturbations [3]. Numerous methods, such as microcontact printing, microfluidic patterning, and photolithography, have been employed to create micropatterned surfaces containing adhesive and non-adhesive regions for cells [4–7]. For example, poly(ethylene glycol) (PEG) photolithography was employed to fabricate arrays of microwells composed of PEG hydrogel walls and glass attachment pads, which was further modified with cell-adhesive ligands to enable formation of high-density leukocyte arrays on glass [8]. These approaches are limited to adherent cells and additional surface chemistry procedures are often required. Alternative methods that do not require adherent cells, including dielectrophoresis [9], optical tweezers [10] and selective dewetting [11], have been adopted for trapping single cells. However, these methods are not suitable for high-throughput applications. Microfluidic devices offering the integration of a variety of methods for single-cell analysis on lab-on-a-chip systems have been reviewed in the literature [12,13]. A two-phase liquid system for measuring messenger RNA expression of specific genes both from total RNA and cells encapsulated in droplets was proposed [14]. The time for analysis with smaller sizes and volumes is expected to decrease dramatically due to the shorter diffusion distances. Nevertheless, the disadvantage of this approach lies in the difficulty with controlling precisely the size of droplets formed. Cells could be entrapped by arrays of dams [15] or structures in microfluidic channels as they move through the microchannels under hydrodynamic force. A poly(dimethylsiloxane) (PDMS) microfluidic platform for parallel single-cell analysis was reported to trap cells individually in dedicated pockets, and thereafter, a number of invasive or non-invasive analysis schemes were performed [16]. Chung *et al.* [17] proposed a microfluidic platform utilizing the hydrodynamic flow in conjunction with a careful disposition of the cell traps in an array formed by a serpentine channel for single-cell capture, stimulation, and imaging. These methods have merits when existing cell assays are integrated onto a microchip platform. However, the status of stress-activated signaling pathways of cells docked in dedicated locations needs to be examined [18]. The strategy of passively confining cells inside microwells which has been reviewed by Lindström and Andersson-Svahn [19] becomes attractive due to its simplicity and ease of implement. The biosensor array has been created by randomly dispersing cells into a microwell array fabricated at the distal tip of an optical imaging fiber, demonstrating the ability to combine the structure of etched optical imaging fibers with fluorescence assay techniques to create a cell-based biosensing system [20]. The cell retainer, a densely packed two-dimensional arrangement of hexagonal picolitre wells, was designed to contain a single untethered cell [21]. The extremely sharp edges of the walls (less than 0.1 μm wide) were designed to make the precipitating cells to settle inside the wells rather than in between; however, this increased the difficulty of fabrication. Moreover, it may be difficult to retrieve target cells from wells without disturbing the surrounding cells. The silicon-based microwell array chip for analyzing the cellular responses of individual cells was fabricated by using a micromachining technique [22]. Furthermore, the relationship between the spacing of the microwells and number of arrayed cells was also addressed that the number of arrayed cells decreased while increasing the spaces between the microwells. Rettig and Folch [23] developed and optimized a simple method for trapping single cells in large open-top microwell arrays. The parameters that maximize single-cell occupancy for two cell types, including the microwell diameter, microwell depth, and settling time, were investigated. A high-density array of SU-8 patterned microwells reported by Chin *et al.* [24] was integrated into a fluidic chamber to guide deposition of adult neural stem cells and confine their progeny. A flow method that enables single-cell trapping in microwells with a size sufficient to allow attachment and division of captured cells was proposed by Park *et al.* [25]. In their work, triangular microwells were found to be most efficient for single cell trapping (about 62% of microwells were filled with single cells) while providing enough space for cell growing and spreading. A passive microfluidic scheme employing hydrodynamic guiding structures in a microwell array was proposed and has a capturing efficiency more than 80% [26]. The implemented structure split the incoming fluid into two pathways having different hydrodynamic resistances and has a capacity to place individual cells into separated microwells for monitoring on cellular behavior at the single-cell level.

Various optical [27], mechanical [28], electric [29], and chemical [30] approaches for cell lysis have been proposed [31]. He *et al*. [32] proposed a method that combines optical trapping and microfluidic-based droplet generation for encapsulating single cells within a picolitre-size aqueous droplet. The trapped cells are lysed rapidly using a YAG laser with a 5-ns pulse duration. Continuous analysis of two dyes loaded into single mammalian cells using laser-based lysis combined with the electrophoretic separation of cell content was achieved using microfluidic chips [33]. The cells are mechanically lysed owing to a cavitation bubble generated by a single laser pulse from a 532-nm picosecond pulsed laser. Laser-mediated lysis is well suited for integration into microfluidic chip platforms; however, it requires complex experimental setups. McClain *et al*. [29] developed an integrated microfluidic device that automatically transports cells to an electrical lysis location. The cell lysate is injected into a separation channel and the labeled lysate contents are electrophoretically separated prior to laser-induced fluorescence (LIF) detection. A continuous electrical cell lysis device in which the width and length of the microchannel changes to generate a focused high electric field strength for cell lysis was proposed [34]. A low electric field strength for the transport of samples at a low operational voltage is employed. The device generates a high electric field strength of 1.2 kV/cm at the orifice to disrupt red blood cells with a 100% lysis rate under an operational voltage of 50 V. Mernier *et al*. [35] proposed a device capable of electrical cell lysis and the evaluation of lysis efficiency in continuous flow using dielectrophoretic cell sorting. An AC electrical field is used at a frequency that optimizes cell lysis while avoiding the creation of bubbles at the electrode surface; the AC field causes a dielectrophoretic effect on the cells that can be used to increase the transit time of the cell in the lysis region. Electric lysis can rapidly lyse a single cell; however, electrophoresis separation, which commonly follows electric lysis, makes continuous injection very difficult [27].

In chemical lysis, a detergent is usually introduced into a cell membrane to create pores within the membrane and lyse the cell. Detergent lysis is well established for bulk biochemical assays. Single-cell capture by a dam-like structure and chemical lysis inside a closed volume was demonstrated in a microfabricated device [30]. Following cell lysis, a limited and stable dilution of intracellular components is used to simplify the requirements for downstream assays. Huang *et al*. [36] designed a microfluidic device to trap cells using a pair of valves. Then, the chamber, where the cell is immobilized, is filled with lysis buffer containing fluorescent antibodies for labeling proteins to quantify the protein contents of a single cell using single-molecule fluorescence counting. Adherent cells were analyzed serially using detergent lysis followed by capillary electrophoresis [37]. Electrophoretic buffer containing sodium dodecyl sulfate (SDS), which is a strong ionic detergent that achieves cell lysis on the order of seconds, was then introduced using sheath flow around the capillary inlet. A simple single-cell lysis method that uses a dense array of microwells (10–30 pL in volume) fabricated from PDMS and a commercially available cell lysis reagent was developed [38]. After the cell lysis solution diffuses into a microwell from the flow cell, which is between a bottom coverslip and a top cell-trapping PDMS sheet separated by two strips of double-faced adhesive tape, the PDMS sheet is rapidly pressed against the bottom coverslip to close each microwell, thereby causing gradual cell lysis. Although chemical lysis is a relatively slow technique, it is very simple and economical. It is also an excellent method for proof-of-concept studies. In the present study, microfluidic chips with microwells, 20 and 30 μm in diameter, are developed for cellular patterning using a flow method and the feasibility of chemical lysis for human carcinoma cells (HeLa cells) at the single-cell level is demonstrated. Compared to the microdroplet-based method [14], the proposed approach of passively confining cells inside microwells is simpler to implement. Cells flowing through a microchannel will gradually settle toward the bottom surface due to gravity and follow streamlines leading into the microwells while losing velocity. Hence, the issue of stress-activated signaling pathways of cells in the methods of the hydrodynamic-based single cell positioning [16,17] could be avoided herein. The present microfluidic chip with microwells is created by soft lithography, and has the additional advantage that it is low-cost and easy to fabricate. However, it has the disadvantage that some cell lysate might dissipate in the proposed microchips. The effects of parameters such as the size of the microwells and the injection flow rate on cellular occupancy are also investigated.

## 2. Materials and Methods

### 2.1. Fabrication of Microfluidic Chips with Microwells

**Figure 1.**
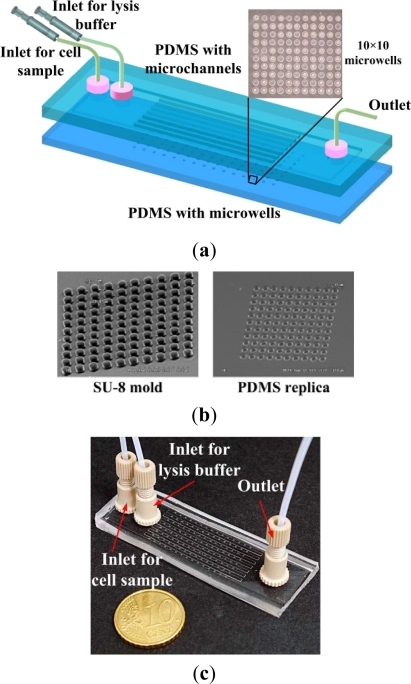
(**a**) Schematic diagram of the proposed microfluidic chip for single-cell-based microarrays; (**b**) SEM micrographs of the SU-8 mold on the silicon wafer and PDMS replica; (**c**) Photograph of the completed microfluidic chip with tubing.

A biocompatible material, PDMS, was adopted for single-cell-based arrays in the microfluidic chip, as illustrated in Figure 1(a). The main channel, formed on the top PDMS layer, is 15 mm wide, 160 μm in height and 65 mm long. The main channel is divided into eight microchannels, each 1 mm wide and 45 mm long, at the center region. Each microchannel contains fifteen 10 × 10 microwells, 20 μm or 30 μm in diameter and 20 μm deep, on the bottom PDMS layer. The mold masters were fabricated by spinning SU-8 (SU-8 50, MicroChem Corp., Newton, MA, USA) on a silicon wafer to define the microwells and microchannel, respectively. The mold master of the microfluidic channels (around 160 μm in height) was fabricated by spinning SU-8 at 500 rpm for 20 s and then at 800 rpm for 35 s on the silicon wafer. The resist was soft baked on a hotplate at 65 °C for 10 min and then at 95 °C for 30 min. The resist was then allowed to cool to room temperature. The SU-8 was exposed to ultraviolet (UV) radiation at a dose of 200 mJ/cm^2^. The post-exposure baking was done at 65 °C for 3 min and then at 95 °C for 10 min. The exposed samples were developed with SU-8 developer for 5 min. The mold master of the microwells (around 20 μm in height) was fabricated by spinning SU-8 at 500 rpm for 20 s and then at 4,500 rpm for 35 s on a silicon wafer. The resist was developed with SU-8 developer for about 2 min after baking and exposure to UV radiation under the conditions mentioned above. PDMS prepolymer mixture (Sylgard-184 Silicone Elastomer Kit, Dow Corning, Midland, MI, USA) was poured and cured on the mold masters to replicate the patterned structures. Scanning electron microscopy (SEM) images of the SU-8 mold with microwells on the silicon wafer and PDMS replica are shown in Figure 1(b). After peeling off the PDMS replica with the microchannel, the inlet and outlet ports were made by a puncher. The two PDMS replicas were bonded after treatment with oxygen plasma in an O_2_ plasma cleaner (model PDC-32G, Harrick Plasma Corp., Ithaca, NY, USA). A photograph of the completed microfluidic chip with tubing is shown in Figure 1(c).

### 2.2. Cell Treatment and Lysis Buffer

A human cervical carcinoma cell line (HeLa cells, ATCC number CCL-2) was cultured for an experimental demonstration of single-cell lysis using the proposed microfluidic chips with microwells. The cells were serially passaged as monolayer cultures in DMEM medium (Gibco, Grand Island, NY, US), with 3.7 g of NaHCO_3_ per liter of medium added, supplemented with 10% fetal bovine serum (FBS, Gibco) and 1% penicillin/streptomycin (Gibco). The cell culture dish (Falcon, Franklin Lakes, NJ, US) was incubated in a humidified atmosphere containing 5% carbon dioxide at 37 °C; the medium was replaced every 1 to 2 days. Cells grown to sub-confluence were washed with phosphate-buffered saline (PBS, Biochrome, pH 7.4) and harvested by a 5-min treatment with 0.25% trypsin and 0.02% ethylene diamine tetraacetic acid (EDTA) (Sigma, St Louis, MO, US). The cells were stained using a standard fluorescence assay with calcein AM (Molecular Probes, Eugene, OR, USA) prior to the experiment. Calcein AM is a green fluorescent dye which is able to penetrate the cell membrane into the cytosol and transform into a fluorescent form when it is hydrolyzed by esterases located inside cells. The cell samples were then suspended in DMEM medium. Cell lysis buffer containing 25 mM Tris (pH 8), 150 mM NaCl, 1 mM EDTA, 1 mM ethylene glycol tetraacetic acid (EGTA), 1% (v/v) Triton X-100, 2.5 mM sodium pyrophosphate, 1 mM β-glycerophosphate, and 2 mM phenylmethylsulfonyl fluoride (PMSF) was employed to lyse single cells deposited in the microwells.

### 2.3. Experimental Procedure

The microchannels were filled with PBS buffer using a syringe pump (Model KDS 101, KD Scientific Inc., Holliston, MA, USA). The trapped bubbles within the microwells were removed using an ultrasonic vibrator. 118 μL of the cellular sample with a concentration of 10^7^ cells/mL was injected using a syringe pump at an inlet flow rate of 2.8 to 4 μL/min, and then a sample of cells with a lower concentration (10^6^ cells/mL) was injected to fill the vacancies of the microwells. Besides, the injection of a sample with a lower concentration is to avoid multiple occupancy. The microfluidic channel was then washed with PBS buffer at a flow rate of 10 μL/min for 1 h. To enhance cellular occupancy, the procedure of injecting the cells sample with a lower concentration mentioned above was repeated four times. Finally, the cellular deposition in microwells was observed and recorded by an inverted fluorescence microscope (model CKX41, Olympus, Tokyo, Japan) mounted on a CCD camera (DP71, Olympus, Tokyo, Japan) and controlled by a computer with Olympus DP controller image software. The fluorescent images were quantitatively analyzed using NIH ImageJ (National Institutes of Health, Bethesda, MD, USA) to measure the intensity of fluorescence. The NIH-Image software has the ability to assess the density of each pixel.

## 3. Results and Discussion

**Figure 2.**
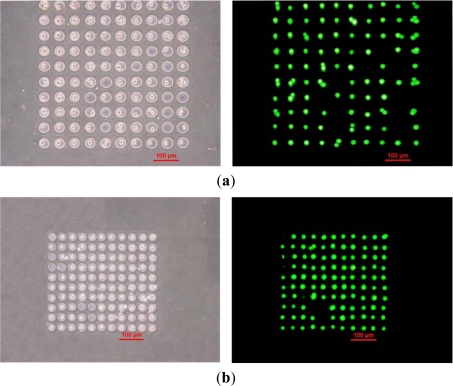
Bright and fluorescence images of micropatterned HeLa cells in microwells with diameters of (**a**) 30 μm and (**b**) 20 μm. The injection flow rate of the cell sample is 2.8 μL/min.

Since the diameter of a suspended HeLa cell is approximately 10 μm, the microwells for depositing cells were designed to be 20 or 30 μm in diameter. Micropatterned HeLa cells in the microfluidic chips with 20- and 30-μm-diameter microwells are shown in Figure 2; the injection flow rate of the cell sample is 2.8 μL/min. The fluorescent images of cells stained by calcein AM demonstrate the viability of the cells. The occupancy of cells in the microwells is higher than 80%.

**Figure 3.**
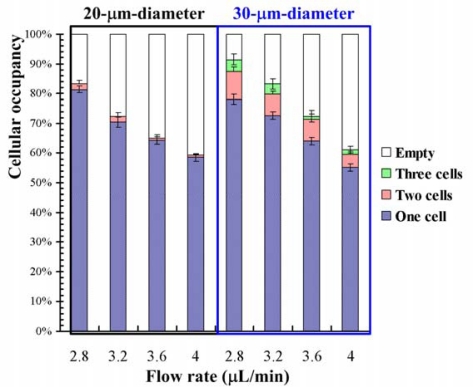
Distributions of 20-μm-diameter and 30-μm-diameter microwell occupancies for HeLa cells for various injection flow rates. The experimental data are based on manual counts of cells in three arrays of 10 × 10 microwells. Each experimental data point represents the average value and the error bar shows the standard error from the mean.

The experimental data of cell occupancy for HeLa cells in the 20- and 30-μm-diameter microwells for various injection flow rates are plotted in Figure 3.

**Figure 4.**
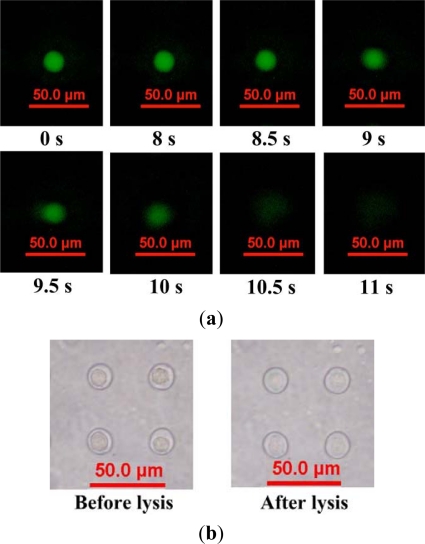
(**a**) Fluorescence images after the introduction of lysis buffer; (**b**) Bright-field images of a single HeLa cell before and after lysis.

The experimental data is based on manual counts of cells in three arrays of 10 × 10 microwells using the inverted fluorescence microscope. Each experimental data point represents the average value, and the error bar shows the standard error from the mean. The occupancy of cells decreases with increasing injection flow rate for both types of microfluidic chip. The cellular occupancy in the 30-μm-diameter microwells (91.45%) was higher than that in the 20-μm-diameter microwells (83.19%) at an injection flow rate of 2.8 μL/min. Most of the occupied 20-μm-diameter microwells contain individual cells, as shown in Figure 3. Some of the 30-μm-diameter microwells contain two or three cells. A 20-μm diameter was thus used for single-cell-based lysis in the present work. Fluorescent micrographs showing fluorescence intensity of calcein AM-loaded HeLa cells after the injection of cell lysis buffer are shown in Figure 4(a). The fluorescence images indicate that the cell membranes were gradually lysed as the lysis buffer was injected. Calcein leakage occurs when cell membranes are lysed; therefore, the fluorescence intensity within the cell decreases after the cell starts to lyse. The cell was fully lysed after 12 s, as shown in Figure 4(a). The bright-field images of the cell before and after lysis are shown in Figure 4(b). The cell membrane was lysed by the detergent-based buffer. The cells remained in the microwells after they were fully lysed.

**Figure 5.**
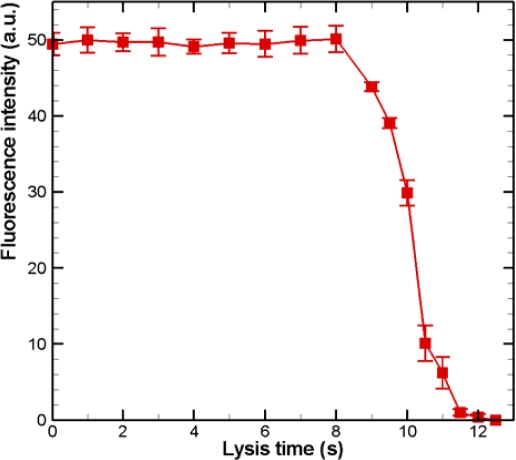
Fluorescence intensity of a single HeLa cell *versus* time after the introduction of lysis buffer. The experimental data are based on measurements of fluorescence in at least three individual cells. Each experimental data point represents the average value and the error bar shows the standard error of the mean.

The fluorescence intensities within single cells were quantified using ImageJ software. The intensity of cells *versus* time is plotted in Figure 5. The experimental data are based on measurements of calcein intensity in at least three individual cells. Each experimental data point represents the average value, and the error bar shows the standard error from the mean. The intensity of calcein drops dramatically at 8 s. The fluorescence intensity decreases to almost zero at 12 s after the injection of the lysis buffer. The fluorescence intensity for the non-lysed cells remains constant during the experiments (data not shown); therefore, the effect of photobleaching is ruled out. Single-cell-based lysis by a chemical buffer in a microfluidic device with microwells is thus feasible.

The present approach of passively confining cells inside microwells is simpler to implement and easier to integrate compared to the microdroplet-based method proposed by Mary *et al.* [14]. As a cell flows through a microchannel, it will gradually settle toward the bottom surface due to gravity and follow streamlines leading into the microwells while losing velocity. Therefore, the issue of stress-activated signaling pathways of cells in the microchips of the flow-based single cell positioning [16,17] could be avoided. The aforementioned approaches of patterning single cells in microwells for single cell analysis [28,38] required manual handling which was not reliable. Microfluidic chips with microwells created by soft lithography are developed in this study, making it low-cost and easy to fabricate.

## 4. Conclusions

Microfluidic chips with microwells were fabricated herein. The occupancy of cells in the 30-μm-diameter microwells was higher than that in the 20-μm-diameter microwells. The occupancy of cells decreased with increasing injection flow rate for both types of microfluidic chip. Most of the occupied 20-μm-diameter microwells contained individual cells. The results of chemical lysis experiments at the single-cell level indicate that the cell membranes were gradually lysed as the lysis buffer was injected; the cell was fully lysed after 12 s. The bright-field images of the cell before and after lysis indicate that the cell membrane was lysed by the detergent-based buffer. However, some cell lysate might dissipate in our microchips. The procedures of encapsulating cell lysate inside the microwells, for example, pressing the top PDMS sheet against the bottom one by microfluidic valves, should be investigated in the future. The proposed microfluidic chips are suitable for high-throughput cell lysis and subsequent single-cell analysis, such as single-cell PCR, culturing, responses and imaging.
